# First person – Deng-Tai Wen

**DOI:** 10.1242/bio.053231

**Published:** 2020-05-29

## Abstract

First Person is a series of interviews with the first authors of a selection of papers published in Biology Open, helping early-career researchers promote themselves alongside their papers. Deng-Tai Wen is first author on ‘Endurance exercise protects aging *Drosophila* from high-salt diet (HSD)-induced climbing capacity decline and lifespan decrease by enhancing antioxidant capacity’, published in BiO. Deng-Tai conducted the research described in this article while a PhD student in the sports science-exercise, aging and genetics lab at Ludong University, Yantai, China, investigating the role of exercise in medicine.


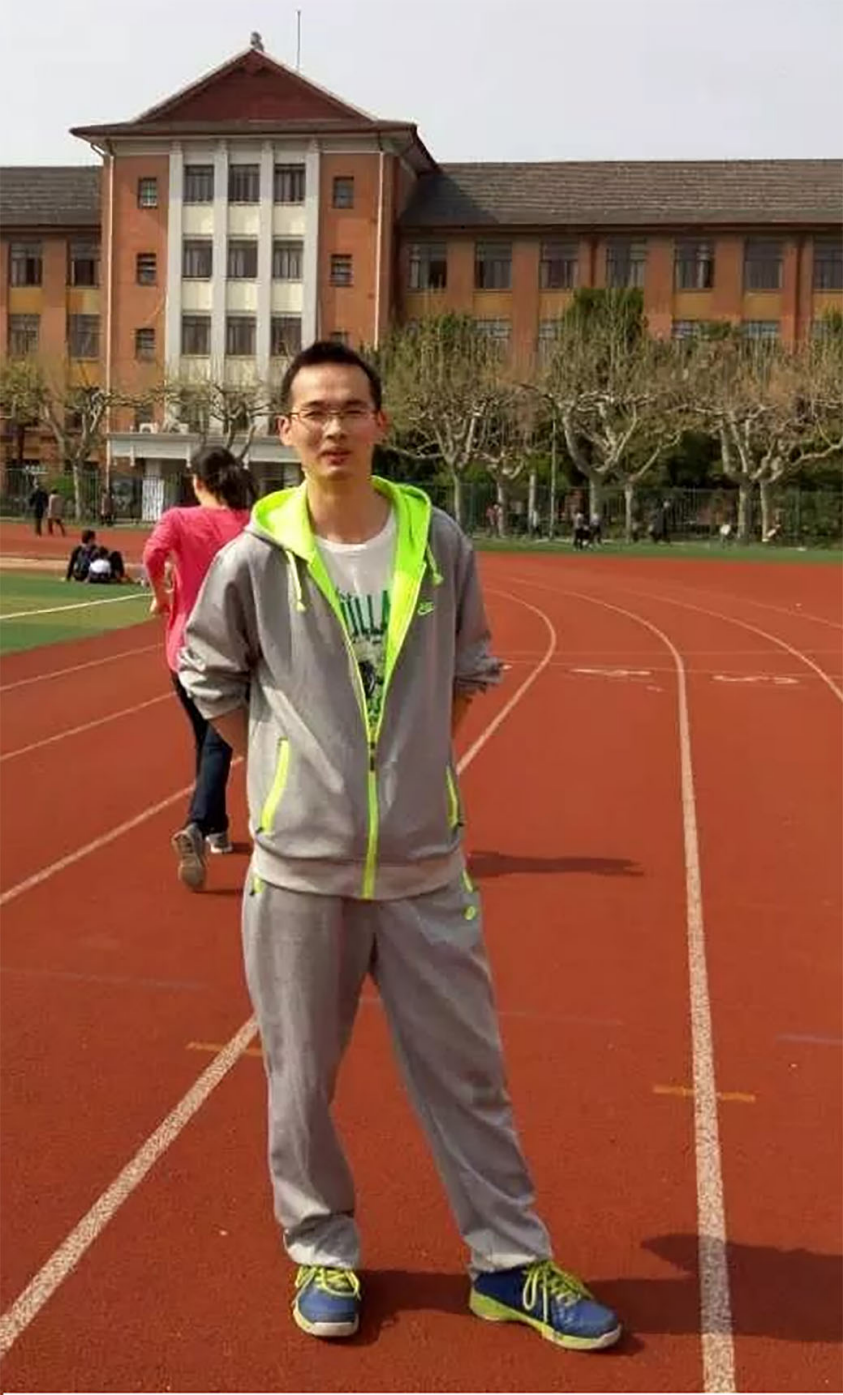


**Deng-Tai Wen**

**What is your scientific background and the general focus of your lab?**

My scientific background is in sports science, and the general focus of my lab is exercise, aging and genetics.

**How would you explain the main findings of your paper to non-scientific family and friends?**

In humans, as in fruit flies, excessive consumption of salty foods can cause disease and death, but exercise can protect us from disease and death induced by salty food.

**What are the potential implications of these results for your field of research?**

I think that it could lead to more genes and molecular pathways associated with exercise promoting health being discovered.

**What has surprised you the most while conducting your research?**

What has surprised me the most is that genetic factors play a regulatory role in exercise adaptation.

**What, in your opinion, are some of the greatest achievements in your field and how has this influenced your research?**

Exercise and nutrition play an important role in the regulation of aging, while heredity plays a key role in this process. I will gradually move from the study of overall aging to the study of tissue and organ aging.

**Figure:**
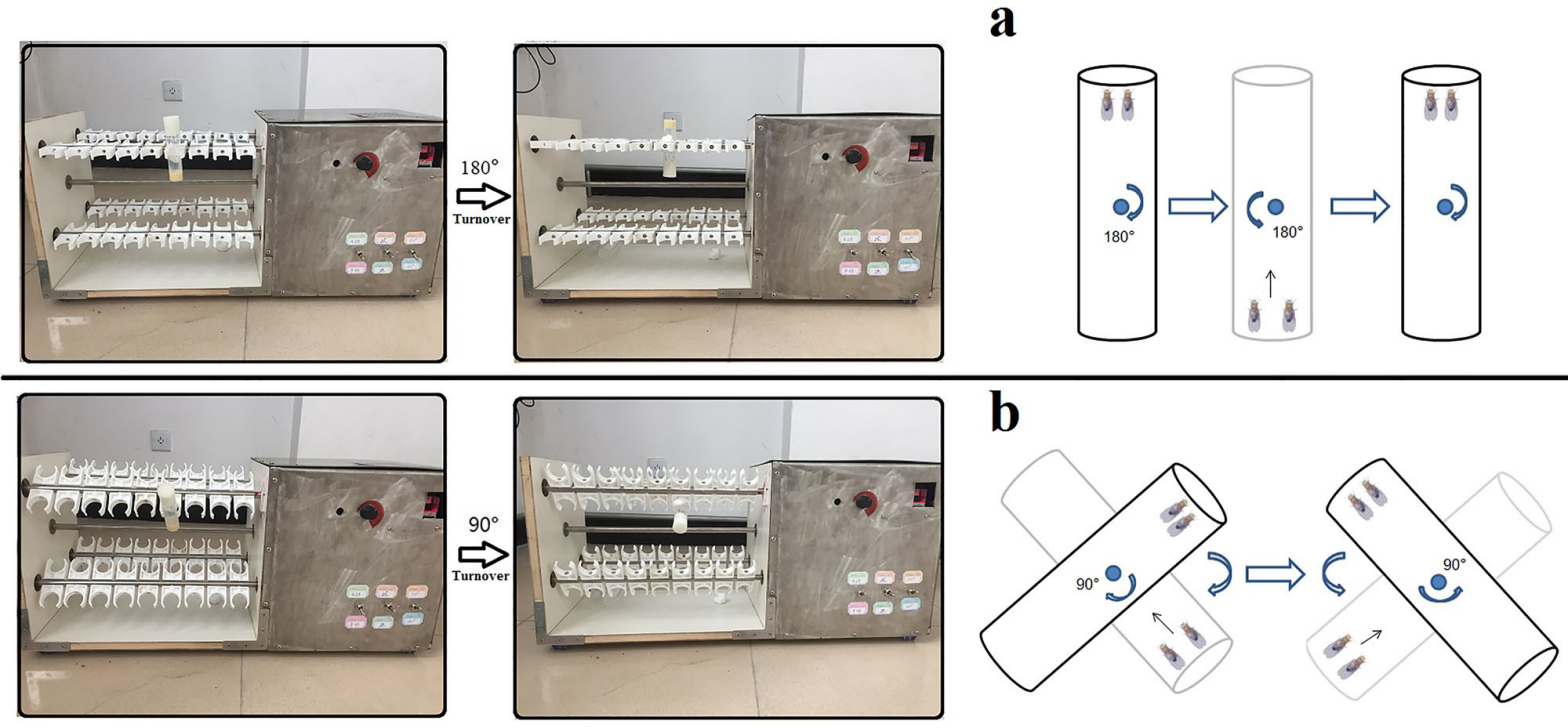
**Exercise training device.** (A) For young and adult flies, vials were vertically loaded in the exercise device, and rotated 180° to make flies constantly climb (just as Power Tower, overcoming weight = total body weight). (B) For aged flies (categorised as 4-week to 5-week-old flies in this study), vials were loaded in the exercise device, their long axis is at an angle of 45° to the horizontal plane (overcoming weight = total body weight×sin45°). When aged flies climbed and reached the top of vial, the vial was rotated 90° to make flies constantly climb.

**What changes do you think could improve the professional lives of early-career scientists?**

I think it's important for early-career scientists to make sure that they remember that practice and taking care while conducting experiments will lead to many new discoveries.

**What's next for you?**

Following on from my work in this paper, I am looking at researching the roles of the *salt* gene and exercise in the aging processes of the heart, skeletal muscle and the brain.
